# Spin to orbital light momentum conversion visualized by particle trajectory

**DOI:** 10.1038/s41598-019-40475-z

**Published:** 2019-03-11

**Authors:** Alejandro V. Arzola, Lukáš Chvátal, Petr Jákl, Pavel Zemánek

**Affiliations:** 1 Instituto de Física, Universidad Nacional Autónoma de México, Apartado Postal 20-364, Ciudad de México, 01000 Mexico; 2 Institute of Scientific Instruments of the Czech Academy of Sciences, Královopolská 147, 612 64 Brno, Czech Republic

## Abstract

In a tightly focused beam of light having both spin and orbital angular momentum, the beam exhibits the spin-orbit interaction phenomenon. We demonstrate here that this interaction gives rise to series of subtle, but observable, effects on the dynamics of a dielectric microsphere trapped in such a beam. In our setup, we control the strength of spin-orbit interaction with the width, polarization and vorticity of the beam and record how these parameters influence radius and orbiting frequency of the same single orbiting particle pushed by the laser beam. Using Richard and Wolf model of the non-paraxial beam focusing, we found a very good agreement between the experimental results and the theoretical model based on calculation of the optical forces using the generalized Lorenz-Mie theory extended to a non-paraxial vortex beam. Especially the radius of the particle orbit seems to be a promising parameter characterizing the spin to orbital momentum conversion independently on the trapping beam power.

## Introduction

Laser beams may spatially confine and drive dielectric particles via so called optical forces following the intensity, phase and polarization profile of the electromagnetic field^1,2^. In this paper we deal with a vortex beam which is focused and resembles a bright doughnut intensity profile with a characteristic helical wave front^3^. Depending on the topological charge $\ell$, vortex beams carry axial orbital angular momentum density (OAM) ($\ell$*ħ* per photon)^4,5^ which can be transferred upon an illuminated particle resulting in an optical torque acting on the particle^6–10^. Vortex beams have been used to initiate rotation of absorbing microscopic particles^11–13^, orbiting of dielectric spheres^14–16^, and spin of non-spherical objects^17,18^.

Besides the orbital angular momentum, a circularly polarized beam can carry axial spin angular momentum (SAM) *σħ* per photon, with *σ* = +1 for right hand circular polarization (RH) or *σ* = −1 for left hand circular polarization (LH). Transfer of SAM to non-spheres, absorbing or birefringent sphere results in spinning of microobjects. It has found applications in rheology as optically driven pumps^19–23^, as a tool quantifying the properties of fluids or gases from a microscopic point of view^19,24–27^, navigating nerve fiber ^growth28^ or as a probe in a new generation of photonic force microscopes^29^ with the ability to detect tiny external torques in addition to tiny external forces^30–32^.

Obviously an elliptically polarized vortex beam carries both types of angular momenta. In the paraxial approximation, the total axial angular momentum density of the beam is done as the sum $(\ell +s)\hslash$ ^4,13^, with *s* being the density of circular polarization or spin density, which is given in terms of the energy densities in the circular basis, I_+_ and I_−_, by *s* = (I_+_ − I_−_)/(I_+_ + I_−_). However, when the beam is tightly-focused, i.e. non-paraxial, a small amount of the spin angular momentum is converted into the orbital one^33–35^ and all azimuthal, radial, and axial components of the optical force depend on this spin-orbit interactions. Since this phenomenon is possible to observe only at the focal volume of the beam, we placed a dielectric particle into the focus and detected the particle trajectory coming from its interaction with the tightly-focused elliptically-polarized vortex beam. We analyzed the radial equilibrium particle position, which reflected the radial force influence, and the particle orbiting frequency proportional to the azimuthal force. Both reveal measurable influence of the spin-orbit conversion. Up to our best knowledge this is the first precise quantitative experimental and theoretical study how the radius of the particle trajectory and its orbiting frequency in a vortex beam is influenced by the polarization and topological charge of the beam.

## Geometry and Experimental Arrangement

**Figure 1:**
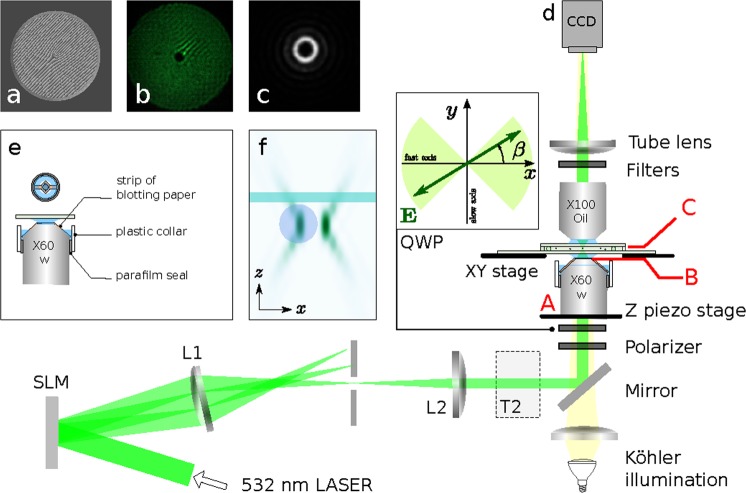
Geometry of the problem and experimental setup. (**a**) An example of the phase mask imposed on the SLM. (**b**) An example of the first order diffraction beam profile at the SLM plane. (**c**) Focused beam imaged on the CCD. (**d**) The opto-mechanical setup of the experiment. The laser beam from Verdi V6 (wavelength *λ* = 532 nm, input power 6W) is expanded 8× by the telescope T1 (not shown in the figure) (*f* = 25 mm, Thorlabs AC127-025-A and *f* = 200 mm, Thorlabs AC254-200-A) and reflected from the spatial light modulator (SLM, Hamamatsu PAL-SLM, X8267-5080DB), passes through the achromatic doublet L1 (focal length *f* = 400 mm, Thorlabs AC254-400-A), aperture that blocks all diffraction orders except the first one, achromatic doublet L2 (*f* = 250 mm, Thorlabs AC254-250-A), telescope T2 (0.75X, with lenses *f* = 200 mm, Thorlabs AC254-200-A and *f* = 150 mm, Thorlabs AC254-150-A), polarizer (linear film polarizer with high extinction ratio and laser damage threshold, Thorlabs LPVISB050), quarter wave plate (QWP, multi-order quarter-wave plate,Thorlabs WPMQ05M-532), high-numerical aperture microscope objective (Olympus UPLSAPO 60× water immersion, NA = 1.2). (**e**) Detail how the objective is covered by a home made plastic collar to prevent water evaporation. The objective is mounted on Z piezo controller (Mad City Labs, NanoF-200, accuracy *δz* = 20 nm), and the sample cell is mounted on XY positioning stage (Prior Scientific, Proscan II). (**f**) A polystyrene particle (Bangs Laboratories, diameter 0.994 *μ*m, refractive index *n*_*p*_ = 1.59) is dispersed in water (*n*_*m*_ = 1.332), pushed by the beam against the top interface of the sample cell and orbits in the focused vortex beam. It is monitored by the microscope objective (Olympus UPLSAPO, 100×, oil immersion, NA = 1.40), lens tube L3 (Achromatic doublet with *f* = 250 mm, Thorlabs AC254-250-A) and CCD camera (Basler acA640-100 gm) providing the sampling time of 2.8 ms and resolution of 40.5 nm/pixel. Capital letters A, B, C denote the planes placed at the rear focal plane of the objective, right behind the objective lenses, and in the focus of the objective, respectively.

**Figure 2:**
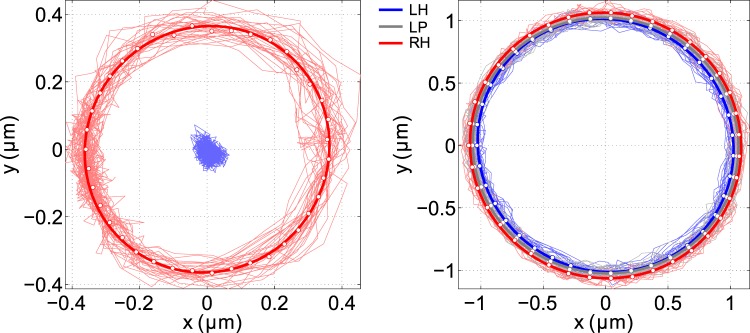
Trajectories followed by the particle center in a vortex beam with topological charge (**a**) $\ell =4$ and (**b**) $\ell =10$ at the xy plane axially placed at the beam waist position and maximal NA = 1.2. The red and blue curves indicate the trajectories for right-handed (RH) and left-handed (LH) circular polarizations, respectively. The gray line in (**b**) records the subtle elliptical shape of the trajectory followed by the particle when the beam is linearly polarized (LP).

**Figure 3:**
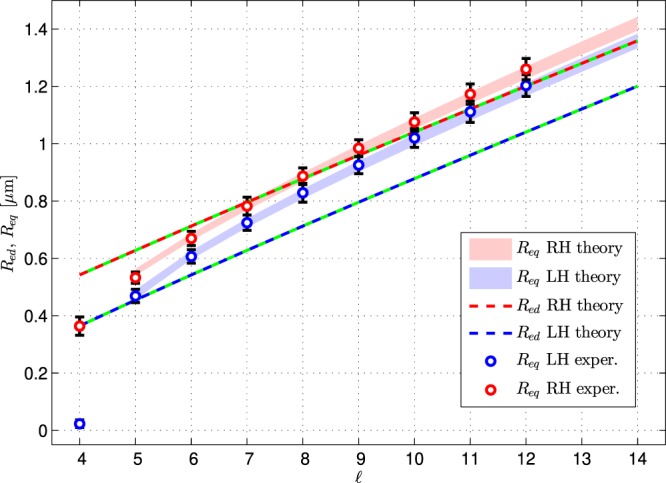
Comparison of the radial position of the maximum of the field energy density *R*_*ed*_ and equilibrium trajectories *R*_*eq*_ of the particle for experimental parameters summarized in Appendix Table A1 at the beam waist at maximal NA = 1.2. Red and blue dots correspond to RH and LH, respectively, the spread of theoretical *R*_*eq*_ corresponds to the spread of the experimental parameters.

A laser beam passes thought the system as Fig. 1d illustrates and enters the sample cell pointing vertically against the gravity. The vortex beam is focused on a diluted solution of polystyrene microparticles dispersed in the sample cell. A particle which enters the beam is pushed by the laser beam against the top glass interface and orbits in the vortex beam (see Fig. 1f). As we show below in Figs 2 and 3, the particle does not orbit at the radial distance corresponding to the highest optical intensity of the vortex beam ring. In contrast to other reported approaches^33^ we cautiously trapped a single particle and used the same particle for the whole parametric study presented below.

A broad Gaussian beam with waist diameter 2*w*_*SLM*_ =17.5 ± 0.5 mm hits the spatial light modulator (SLM) where a particular phase mask is imposed. We use a blazed grating added to the original helical phase at the SLM, giving the characteristic fork dislocation (see Fig. 1a)^36^. The width, intensity and topological charge of the first order diffracted (paraxial) beam are controlled depending on the phase mask extend, modulation depths and vorticity and its lateral intensity profile is shown in Fig. 1b^16,17^. Such a flexibility of the system enabled us to modify the above mentioned beam parameters while we kept the same particle confined. On the top of that we also employed the SLM to compensate optical aberrations in the optical path^17,37,38^.

The elliptical polarization of this paraxial beam is controlled with a quarter-wave plate (QWP) placed close to the entrance aperture of the objective. The linear polarization of the beam incident on the QWP was set precisely along x axis by a high power polarizer. The angle *β* between the x axis and the fast axis of the quarter wave plate is set with a rotatory stage with an accuracy of *δβ* = 1°. The beam is focused by the high numerical aperture water immersion objective to the sample cell. An example of its image on the CCD is shown in Fig. 1c. The water immersion objective suppresses the strong spherical aberrations appearing in oil immersion objectives when the sample is immersed in water several microns far from the cover glass.

## Spin-to-Orbit Angular Momentum Conversion Visualized by Particle Trajectory

The azimuthal force component (see Appendix for details) gives rise to the orbiting motion of the particle around the axis of the beam. It is important to stress that the dynamics of the particle is not deterministic, but in general it is subjected to the thermal fluctuations of the surrounding medium. The dynamics of the particle is recorded with a video-microscopy system providing sampling time of 2.8 ms and resolution 40.5 nm/pixel. The trajectory followed by the centroid of the particle is estimated from the digitalized images with an accuracy of less than 5 nm using the least-squares fitting method^39^. Usually units of mW are enough to maintain the particle on a stable orbit near the top glass against the thermal fluctuations and the gravity force.

Figure 2 shows the trajectories acquired for the same particle in the focused vortex beam with the topological charge $\ell =4$ and $\ell =10$ and for both circular polarizations. For $\ell =4$ (Fig. 2a), when the beam goes from left-handed (LH) to right-handed (RH) circular polarization, there is a significant qualitative change in the particle dynamics, transiting from an equilibrium position in the center of the beam for LH to a stable orbit around it for RH. It is known that the on-axial or off-axial equilibrium position of the particle depends on the ratio of the particle diameter and the radius of the narrowest ring defined by the maximum of the energy density *R*_*ed*_^40,41^. Therefore this observation proves in a very sensitive way the existence of significantly different lateral equilibrium positions for LH and RH circular polarizations^34^. We also observed experimentally that for $\ell  < 4$ the particle is always trapped close the beam axis. For $\ell  > 4$ and any polarization the particle is pushed away from the beam axis to an equilibrium radial position where the particle orbits around the beam center following the optical torque coming from the azimuthal scattering force. We can see already in Fig. 2b a more elliptical trajectory if the polarization is linear. To compare qualitatively the radii of the particle orbit for different beam parameters, we fit an ellipse to the experimental data^42^ denoted by the thick curves in Fig. 2. The white dots in Fig. 2 denote the mean local radius. For simplicity, hereafter, we will denote the mean radius of the circular trajectory *R*_*eq*_ which was determined as the minimal radial distance between the circle and all the particle positions^43^.

Comparison with theoretical results in Fig. 3 demonstrates that *R*_*eq*_ is generally different from the radial positions of the maxima of the field energy density *R*_*ed*_ and its experimental values correspond very well to the theoretical model. For large values of $\ell$ ($\ell \ge 7$) the equilibrium positions of the particle grow approximately linearly with the slope *m* = 0.093 *μ*m, and with a constant gap beneath circular polarizations of 0.058 *μ*m. Later on we will discuss this phenomenon in more details.

**Figure 4:**
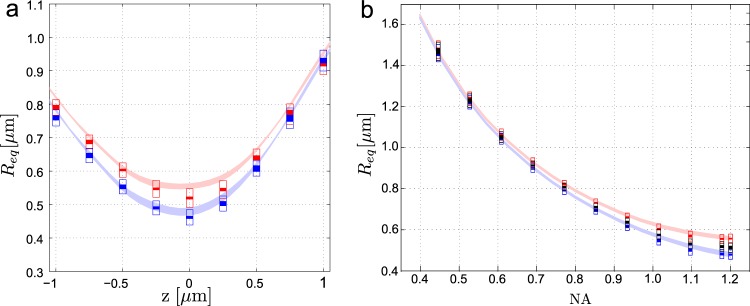
Comparison of measured (rectangles) and calculated (bands) mean orbiting radius *R*_*eq*_ as a function of the z-position of the particle with respect to the beam focus (**a**) and focusing numerical aperture NA (**b**) for the beam with topological charge $\ell =5$ and circular polarizations RH, LH and LP. Blue and red bands correspond to measured data for RH and LH polarizations, respectively. The horizontal lengths of the rectangles indicate the uncertainty of 1 degree in the orientation of the QWP. The vertical lengths of the full rectangles denote the difference between the semimajor and semiminor axes of the fitted elliptical trajectory, while the empty rectangles are related to the mean standard deviation of the Brownian trajectory along the radial direction. The underlying lighter bands account for the transversal stable positions of the particle coming from our theoretical analyses based on Mie-theory for the aperture angles *θ*_max_ = 60–63° capturing the effect of experimental uncertainty in these parameters.

Since the radius of the particle orbit varies with the axial position of the particle in the beam, Fig. 4a illustrates this dependence for the topological charge equal to $\ell =5$ and both polarizations. If the particle *z* positions are close to the beam waist (*z* = 0), the trajectory radii *R*_*eq*_ differ more with the beam polarizations. Let us highlight very good agreement between the theoretical description and the experimental data even though we have used only the parameters obtained from the experiment (see Appendix Table A1) without any fitting. In all the studies presented below we always first measured the axial dependence of the mean radius of the particle trajectory, determined the axial position giving the minimal orbit radius *R*_*eq*_, and set this axial position as *z* = 0 (as in Fig. 4a).

Figure 4b compares experimental and theoretical results of *R*_*eq*_ at *z* = 0 for one selected vortex beam with $\ell =5$, both polarizations RH and LH, and NA going from NA = 0.4 until the maximum value of the objective NA = 1.2. The effective numerical aperture NA is set at the SLM by the diameter of the phase mask, diffracting the incident beam to the first order and thus controlling the diameter of the beam entering the objective^38^. As it is intuitively expected, noticeable differences between *R*_*eq*_ for both polarizations are seen for larger NA where the beam is more tightly focused and stronger spin-orbit coupling occurs. In contrary, negligible influence of the beam polarization on the particle trajectory radius *R*_*eq*_ can be found for NA bellow 0.5.

**Figure 5:**
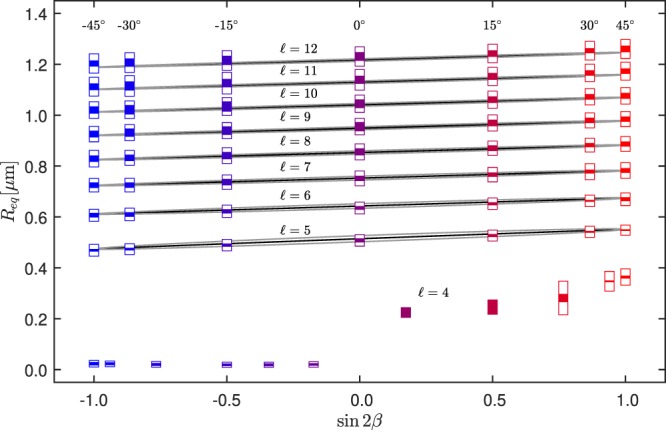
Orbiting radii *R*_*eq*_ as a function of the spin density of the incident beam *s* = sin 2*β* and topological charge $\ell$. The rectangles denote the measured values and the symbolism is the same as in Fig. 4. The values at the leftmost and rightmost side ($s=\pm \,1$) correspond to the case of circular polarizations, LH and RH correspondingly. The full black, or upper and lower gray curves denote Mie-theory calculations of the transversal stable position of the particle at *z* = 0 for experimental parameters (Appendix Table A1) assuming circular trajectory, or semimajor and semiminor axis of elliptical trajectory, respectively.

Figure 5 summarizes our main results as the dependence of the mean radius *R*_*eq*_ of the particle orbits on *s* = sin 2*β* and nine different topological charges from $\ell =4$ to $\ell =12$. The theoretical prediction is plotted in the same graph as black and gray curves. The vertical lengths of the full rectangles in this figure represent again the difference between minor and major semiaxis in the observed trajectory. Apart from $\ell =4$, as we go from *s* = −1 to *s* = 1, for a fixed topological charge, the orbiting mean radius exhibits a linear growth with similar slopes for all the topological charges. To understand this behavior, it is important to realize that part of the energy of the vortex with topological charge $\ell$ entering into the objective is redistributed in the focalized beam in four more vortices with topological charges $\ell \pm 1$ and $\ell \pm 2$ according to the ellipticity (see appendix). For example, for LH this part of the energy is redistributed in two extra vortices with topological charges $\ell -1$ and $\ell -2$ which in turn have smaller sizes than the main vortex with topological charge $\ell$. For RH, the extra vortices have topolical charges $\ell +1$ and $\ell +2$. Owing to this redistribution of energy, it is expected that the radial equilibrium position (*R*_*eq*_) of the particle is not the corresponding to the maximum of intensity of the main vortex ($\ell$), but it will be slightly shifted by the other components. Using highly simplified considerations, described in the appendix, we were able to express the radius of the particle orbit as

1 $${R}_{eq}\approx m\ell +b\,\sin \,2\beta +\delta $$

where *m*, *b* and *δ* are constants. Fitting Eq. (1) with the data shown in Fig. 5 for $\ell \ge 7$, we obtain *b* = (0.029 ± 0.001) *μ*m which is half the value of the total shift in the radial position when the polarization goes from LH to RH. Using the other fitted values, *m* = (0.095 ± 0.004) *μ*m and $\delta =(0.09\pm 0.03)\,\mu \text{m}$, we obtain *b*/*m* = 0.3 giving good agreement with the theoretical value calculated only with the focused-beam model *b*/*m*|_*theory*_ = 0.27 (see Appendix Eqs (14) and (15)). In this context, the ratio *b* sin 2*β*/*m* quantifies the efficiency of spin-to-orbit angular momentum conversion. If the beam is linearly polarized, sin 2*β* = 0 and thus we can conclude there is no spin-to-orbit angular momentum conversion.

## Orbiting Frequency

Due to the mainly non-conservative azimuthal force, the particle orbits and its orbiting frequency can be obtained directly from the experimental time record of the particle motion. Orbiting frequency is power dependent and is also influenced by the particle proximity to the surface via hydrodynamic drag coefficient. Therefore its comparison with the theoretical results is less straightforward comparing to the mean orbit radius *R*_*eq*_.

**Figure 6:**
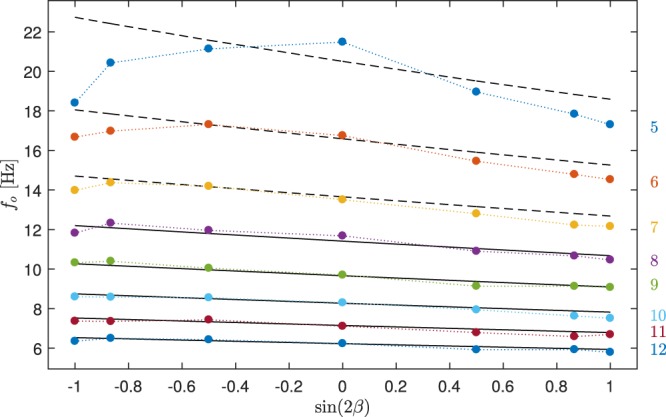
Mean experimental orbiting frequency in colors corresponding to topological charges $\ell$ denoted at right. Full lines give the best coincidence between theory and experiment if the same parameters as in Fig. 5 are used and the theoretical trapping power is adjusted by a factor 1.17 found by fitting. The ratio between found theoretical and experimental power is 1.17. Dashed lines used the same parameters and extrapolates the theoretical results for lower topological charges.

We took two orbits of x particle coordinates and determined the orbiting frequency in this interval by fitting a cosine function. We repeated the procedure for all subsequent non-overlapping data sets corresponding to two orbits. Figure 6 shows the experimental results in colors as the function of sin 2*β*. They are compared with the theoretical results giving excellent agreement for $8\le \ell \le 12$ where the only optimized parameter was the trapping power. Found ratio between optimal theoretical power and used experimental power is 1.17. Even though we implemented in the theory the experimental value of the increased drag coefficient due to the surface proximity (see Appendix Table A1), influence of such hydrodynamic interaction in combination with varying axial optical force due to the retro-reflected beam from the surface can be more complex and only experimental determination of the particle-surface distance could lead to deeper understanding. The non-uniformities observed in the beam profile is another important aspect not included in the theoretical description (see Appendix Fig. A2). Figure 6 presents another example of direct demonstration of spin-orbit interaction in focused vortex beam.

## Conclusion

Up to our best knowledge this is the first comprehensive experimental and theoretical comparison of behavior of a single particle in the non-paraxial vortex beam with arbitrary elliptical polarization. Previous study reported either behavior of several particles in the vortex beam without relevant theoretical analysis^33^ or focused only on the orbiting frequency and linearly polarized beam^16,44^. We were able to control the spin to orbital conversion either by changing the circular polarization of the beam or by reducing the numerical aperture of the focusing system, and study its influence on the behavior of the trajectories of the trapped particles. We showed that for a certain topological charge (*l* = 4 in our case) it is even possible to observe a suppression of the orbiting of the particle if the circular polarization goes from the same sense of the vorticity to the opposite. We also showed that the orbiting trajectories can render elliptical trajectories if the polarization is not circular. We observed influence of the spin to orbital conversion in the radius of the equilibrium orbit and also in the orbiting frequency. The influence was stronger for smaller topological charges. Finally, we found a very good agreement between the experimental results and the theoretical ones which is based on the Lorenz-Mie and Richards-Wolf theories even using the experimentally determined parameters.

## Supplementary information

Appendix

## Data Availability

Data and resources in support of the findings of this study are available from the corresponding authors upon reasonable request.
