# Supplementary material for: Spin to orbital light momentum conversion visualized by particle trajectory
Source: Sci Rep. 2019 Mar 11;9:4127. doi: 10.1038/s41598-019-40475-z (PMC6411984; doi:10.1038/s41598-019-40475-z)
Supplement: Supplementary file 1 — Appendix [file 41598_2019_40475_MOESM1_ESM.pdf]

# Spin to orbital light momentum conversion visualized by particle trajectory

Alejandro V. Arzola, Lukáš Chvátal, Petr Jákl and Pavel Zemánek

# Appendix

## Measurement procedure

Generation of high-quality vortex beam is extremely sensitive to optical aberrations in the optical path and therefore we adopted the *in-situ* correction method[1] to eliminate their influence. All results presented in the paper are performed with the same particle. Once the particle was trapped laterally in the vortex ring the topological charge $\ell$ was set by phase mask at the SLM. The particle started to orbit and the z-piezo stage was moved while the minimal radius of particle orbit $R_{eq}$ was determined. The Z stage was set to the position of minimal $R_{eq}$ and the polarization of the beam was modified by rotation of the QWP with an estimated precision 1 degree. For each QWP 6000 frames were acquired. Then the $\ell$ was increased, the new axial position of the minimal $R_{eq}$ was determined and the measurement was repeated till the $\ell = 12$ was finished.

Since we used water immersion objective and the particles were observed also in water the z-shift of the stage was equal to the shift of the beam focus position[2,3]. The z-stage position was controlled with an accuracy of $\delta z = 20$ nm. The imaging system is well calibrated using calibration grid made by electron litography giving 40.5 nm/pixel. The list of experimental parameters gives Table **A1** below.

## Data processing

The complete set of positions was subject to circle and ellipse fitting with method suggested by Kasa[4], where the sum of radial distances between the circle and all the particle positions is minimized. The stochastic motion of the particle in the radial direction around its equilibrium position $R_{eq}$ has a Gaussian distribution $\propto (2\pi\sigma^2)^{-1/2}\exp[-(R-R_{eq})^2/2\sigma^2]$. Assuming the equipartition theorem, the variance is connected to the radial stiffness $\kappa_r$ at $R_{eq}$ as

$$\sigma^2 = \langle[\Delta R(t)]^2\rangle = \frac{k_B T}{\kappa_r} \tag{1}$$

So the radial stiffness $\kappa_r$ can be obtained either from Eq. (1) or from fitting Gaussian distribution of probability to the experimental profile (see Fig. **A1**d). Using the power spectrum density of radial positions $S(f) = 2|\mathscr{F}\{R(t)\}|^2$[5,6]

$$S(f) = \frac{4\gamma_\parallel\, k_B T}{(2\pi\gamma_\parallel)^2(f^2+f_c^2)} = \frac{k_B T}{\pi^2\gamma_\parallel}\frac{1}{(f^2+f_c^2)} \tag{2}$$

$$= \frac{4\gamma_\parallel\, k_B T}{\kappa_r^2}\frac{1}{1+(f/f_c)^2} = \frac{4\gamma_\parallel\,\sigma^4}{k_B T}\frac{1}{1+(f/f_c)^2} \tag{3}$$

the drag coefficient $\gamma_\parallel$ is related to the ‘corner frequency’ by $\omega_c = 2\pi f_c = \kappa_r/\gamma_\parallel$ (using either $\sigma$ or $\kappa_r$):

$$\gamma_\parallel = \frac{\kappa_r}{2\pi f_c} = \frac{k_B T}{2\pi f_c\sigma^2} \tag{4}$$

where $k_B$ is Boltzman constant and $T$ is thermodynamical temperature. Independently, one can compare with the value $\Delta R(f=0)$ which yields (using either $\sigma$ or $\kappa_r$):

$$\gamma_\parallel = 4k_B T\frac{|\Delta R(f=0)|^2}{\sigma^4}. \tag{5}$$

The hydrodynamic drag coefficient is influenced by particle-surface proximity as $\gamma_\parallel = \gamma_0\psi$ where $\gamma_0 = 6\pi a\eta$ denotes the Stokes formula with $a$ and $\eta$ denoting the particle radius and medium viscosity, respectively, and $\psi$ denotes the Faxen's correction[7–9] to the proximity of the surface

$$\psi(a,h) = \left[1-\frac{9}{16}(a/h)+\frac{1}{8}(a/h)^3-\frac{45}{256}(a/h)^4-\frac{1}{16}(a/h)^5\right]^{-1}. \tag{6}$$

where $h$ is the distance of the particle center from the glass interface. Since we have not measured the particle-surface distance we could not used the above described approach. Instead we determined the corner frequency from the fit of experimental data to $S(f)$ and using the trap stiffness $\kappa_r$ determined from Eq. (1) we obtained the drag coefficient $\gamma_\parallel$. To obtain the correction term $\psi$ we used the expected size of the particle and local viscosity corresponding to local temperature $25^o$ C as

$$\eta(T) = A\times 10^{B/(T-140)} \tag{7}$$

where $A = 2.414E-5K$, $B = 247.8K$, $T$ is in Kelvins. Obtained values are summarized in Table **A1**.

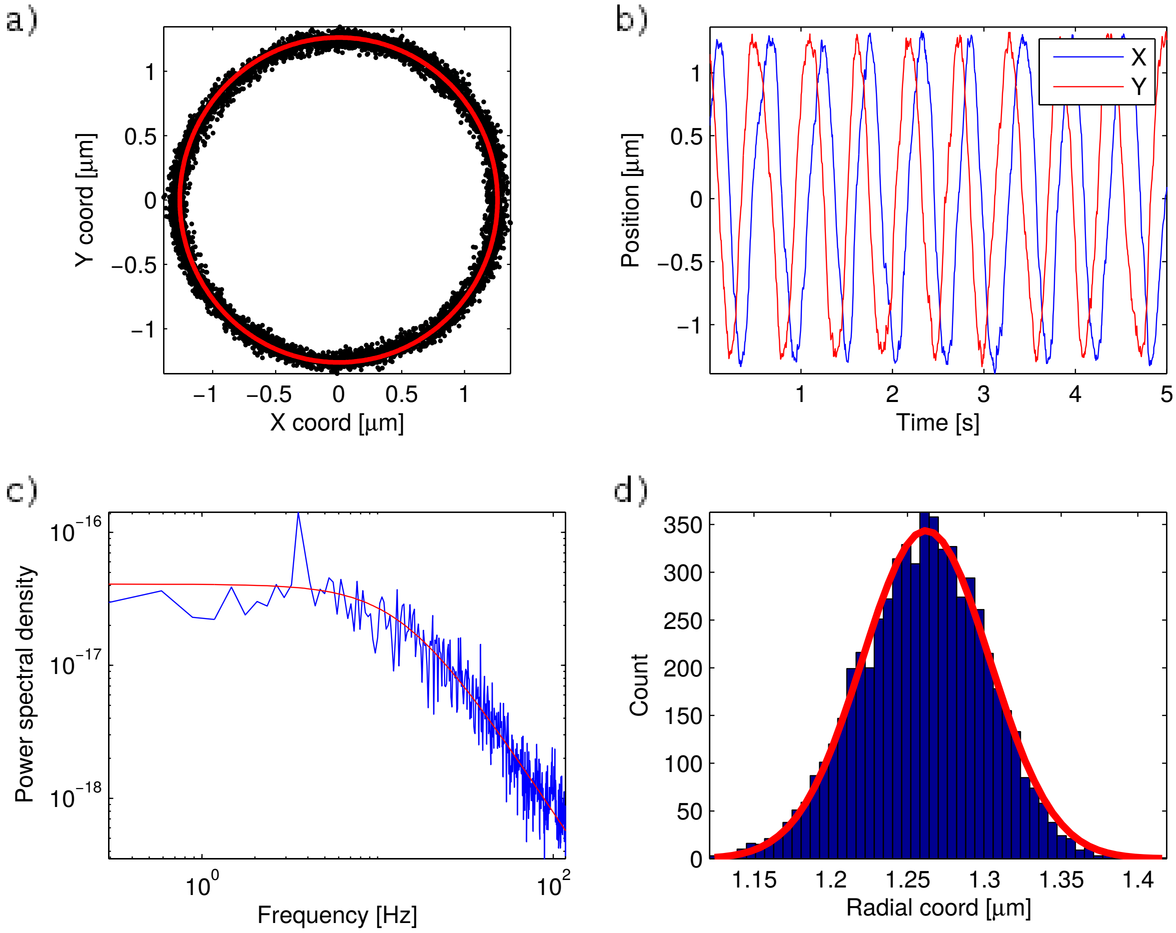


**Appendix Figure A1.** Examples of processed data a) Particle trajectory in lateral plane b) x and y positions of the particle in time c) The power spectral density of radial position of the particle with corner frequency $f_c = 14Hz$ d) Probability distribution of radial position of the particle giving radial stiffness $\kappa_r = 2.4$ N/m.

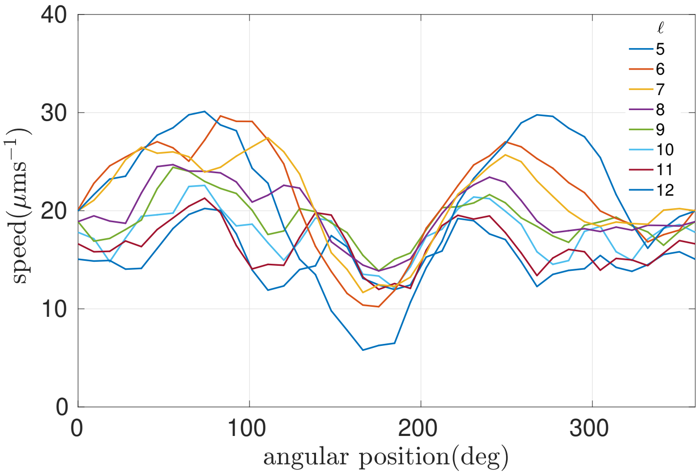


**Appendix Figure A2.** Local speed of the particle as a function of the angular position for different topological charges $\ell$ and for the LH polarization.

| | | | |
|---|---|---|---|
| Particle radius | a=994 nm | Stiffness from histogram | $\kappa_r = 2.4 \cdot 10^{-6}$ N/m |
| Particle refractive index | $n_p = 1.59$ | Corner frequency | $f_c = 14$ Hz |
| Water refractive index | $n_m = 1.332$ | | |
| Vacuum trapping wavelength | $\lambda = 532nm$ | Freespace viscosity | $\eta = 0.9 \cdot 10^{-3}$ Pa·s |
| Temperature | 25 °C | Freespace drag | $\gamma_0 = 8.4 \cdot 10^{-9}$ kg/s |
| Video record frequency | 235 Hz | Drag from $\kappa_r$ | $\gamma_\parallel = 2.7 \cdot 10^{-8}$ kg/s |
| Points in one record | 6000 | Drag from $S(0)$ | $\gamma'_\parallel = 2.6 \cdot 10^{-8}$ kg/s |
| Gaussian beam waist radius $w_{SLM}$ | 8.75 ±0.25 mm | Drag ratio | $\psi = 3.2$ |
| Magnification between SLM and plane A | 0.47 | | |
| Laser beam power at the sample plane | 7.9 ± 0.2 mW | | |
| Transmision of the objective 60x | 0.91 ± 0.02 | | |

**Appendix Table A1.** Parameters of the experiment.

## Beam description

Let us start with a description of the paraxial beam entering the objective at plane A, i.e. at the front focal plane of the objective (Fig. 1d in the main manuscript). Let us assume the monochromatic wave of electric field $\mathscr{E} = \Re[\mathbf{E}\,\mathrm{e}^{-\mathrm{i}\omega t}]$ is described by its complex amplitude $\mathbf{E}$. The vortex beam coming from the SLM is linearly polarized along $x$ axis and its width is done by the extend of the phase grating on the SLM. This beam passes through a quarter wave plate rotated with its fast axis by an angle $\beta$ with respect to the $x$ axis (see Fig.1 in the main manuscript) and its influence is described by the following Jones matrix:

$$Q = \begin{pmatrix} \cos^2\beta + \mathrm{e}^{\mathrm{i}\frac{\pi}{4}}\sin^2\beta & (1-\mathrm{e}^{\mathrm{i}\frac{\pi}{4}})\cos\beta\sin\beta \\ (1-\mathrm{e}^{\mathrm{i}\frac{\pi}{4}})\cos\beta\sin\beta & \mathrm{e}^{\mathrm{i}\frac{\pi}{4}}\cos^2\beta + \sin^2\beta \end{pmatrix} \tag{8}$$

Assuming that the field at plane A (the entrance of the objective) is a perfect image of the field in the SLM, we can describe the incident beam at plane A as:

$$\mathbf{E}_A = E_{A0}\mathrm{e}^{\mathrm{i}\ell\varphi}\mathrm{e}^{-(x_A^2+y_A^2)/w_A^2}\left(E_{Ax}\hat{\mathbf{e}}_x + E_{Ay}\hat{\mathbf{e}}_y\right) = \frac{E_{A0}}{\sqrt{2}}\mathrm{e}^{\mathrm{i}\ell\varphi}\mathrm{e}^{-(x_A^2+y_A^2)/w_A^2}\left(E_{A+}\hat{\mathbf{e}}_+ + E_{A-}\hat{\mathbf{e}}_-\right), \tag{9}$$

where $E_{A0}$ denotes the field amplitude in plane A, $w_A = Mw_{SLM}$ the beam waist radius with the magnification of the relaying optical system $M = 0.47$ between the SLM and plane A. The coordinates $(x_A, y_A)$ correspond to the transversal ones in the plane A. $p$ and $q$ correspond to the RH and LH circular polarization amplitudes, respectively. The RH and LH circular basis is given by $\hat{\mathbf{e}}_\pm = (\hat{\mathbf{e}}_x \pm \mathrm{i}\hat{\mathbf{e}}_y)/\sqrt{2}$, where $\hat{\mathbf{e}}_x$ or $\hat{\mathbf{e}}_y$ denotes unit vector along $x$ or $y$ axis, respectively. Table A2 defines more clearly the used polarization parameters.

The amplitude of the electric field can be expressed in terms of the experimental parameters as $E_0 = \sqrt{2\mu_0 cP/\zeta}$. From Eq.(9), it is easy to show that the difference in energy between the RH and LH polarizations, which defines the spin angular

| Polarization | General<br><br>Relation | Linear<br>right hand<br>LP | Circular<br>left hand<br>RH | Circular<br><br>LH |
|---|---|---|---|---|
| $\beta$ | | $0, \pi/2$ | $+\pi/4$ | $-\pi/4$ |
| $s$ | $\sin 2\beta$ | 0 | 1 | -1 |
| $p$ | $1+\tan\beta$ | 1 | 2 | 0 |
| $q$ | $1-\tan\beta$ | 1 | 0 | 2 |

**Appendix Table A2.** Definition of polarization parameters.

momentum density, is given by:

$$s = \frac{|E_+|^2 - |E_-|^2}{|E_+|^2 + |E_-|^2} = \sin 2\beta. \tag{10}$$

The beam passes through the microscope objective following the Richards and Wolf theory[10] (assuming Fresnel transmission coefficients $t_s = t_p = 1$) the refracted field immediately after the objective (plane B) is expressed in the circular basis as follows[11]

$$\begin{aligned} \mathbf{E}_\infty(\theta,\varphi) &= \frac{E_{A0}\cos\beta \mathrm{e}^{\mathrm{i}\ell\varphi}\mathrm{e}^{-f^2\sin^2\theta/w^2}}{2\sqrt{2}}\frac{\sqrt{\cos\theta}}{\sqrt{n_m}} \times \\ & \left[\left(g(\theta)p - h(\theta)q\mathrm{e}^{-\mathrm{i}2\varphi}\right)\hat{\mathbf{e}}_+ + \left(g(\theta)q - h(\theta)p\mathrm{e}^{\mathrm{i}2\varphi}\right)\hat{\mathbf{e}}_- - \sqrt{2}\sin\theta\left(p\mathrm{e}^{\mathrm{i}\varphi} + q\mathrm{e}^{-\mathrm{i}\varphi}\right)\hat{\mathbf{e}}_z\right], \end{aligned} \tag{11}$$

with $g(\theta) = 1+\cos\theta$, $h(\theta) = 1-\cos\theta$ and $\theta$ is the polar angle.

The field in the beam focus (plane C) is thus a superposition of all the incident waves with angles $\theta$ and $\varphi$ in Eq. (11), following[12,13],

$$\mathbf{E}(\rho,\varphi,z) = \frac{\mathrm{i}kf\mathrm{e}^{-\mathrm{i}kf}}{2\pi}\int_0^{\theta_{\max}}\int_0^{2\pi}\mathbf{E}_\infty(\theta,\varphi)e^{\mathrm{i}\Xi}\sin\theta \mathrm{d}\varphi \mathrm{d}\theta, \tag{12}$$

where $\Xi = kz\cos\theta + k\rho\sin\theta\cos(\varphi-\varphi)$. $(\rho,\varphi,z)$ are the cylindrical coordinates measured from the focal point and $\theta_{\max}$ defines the maximum angle of the cone related to the numerical aperture of the focusing optics NA $= n_m \sin\theta_{\max} = D/(2f)$, $D$ and $f$ denotes the diameter of the input aperture and focal length of the focusing objective, respectively. Regrouping Eqs. (12), one gets

$$\mathbf{E}(\rho,\varphi,z) = W\cos\beta\mathrm{e}^{\mathrm{i}\ell\varphi}\left[\left(A_\ell p + B_{\ell-2}q\mathrm{e}^{-2\mathrm{i}\varphi}\right)\hat{\mathbf{e}}_+ + \left(A_\ell q + B_{\ell+2}p\mathrm{e}^{2\mathrm{i}\varphi}\right)\hat{\mathbf{e}}_- - \mathrm{i}\sqrt{2}\left(C_{\ell+1}p\mathrm{e}^{\mathrm{i}\varphi} - C_{\ell-1}q\mathrm{e}^{-\mathrm{i}\varphi}\right)\hat{\mathbf{e}}_z\right] \tag{13}$$

with

$$\begin{aligned} W &= \frac{E_{A0}}{\sqrt{2n_m}}\frac{\mathrm{i}^{\ell+1}kf\mathrm{e}^{-\mathrm{i}kf}}{2}, \\ A_m &= \langle f_w(\theta)\mathrm{e}^{\mathrm{i}kz\cos\theta}(1+\cos\theta)J_m(k\rho\sin\theta)\sin\theta\sqrt{\cos\theta}\rangle, \\ B_m &= \langle f_w(\theta)\mathrm{e}^{\mathrm{i}kz\cos\theta}(1-\cos\theta)J_m(k\rho\sin\theta)\sin\theta\sqrt{\cos\theta}\rangle, \\ C_m &= \langle f_w(\theta)\mathrm{e}^{\mathrm{i}kz\cos\theta}J_m(k\rho\sin\theta)\sin^2\theta\sqrt{\cos\theta}\rangle, \\ f_w &= \exp\left(-\frac{f^2\sin^2\theta}{w^2}\right), \\ &\text{and} \\ \langle X\rangle &= \int_0^{\theta_{\max}} X\,\mathrm{d}\theta. \end{aligned}$$

The azimuthal-dependent exponential functions indicate that the original vortex with initial topological charge $\ell$ transforms to one in the focal plane composed of several vortex beams with topological charges $\ell$, $\ell\pm1$, and $\ell\pm2$ with amplitudes defined by the circular components $p$ and $q$ and by the polar angle $\theta_{\max}$. The circular components in the tightly focused beam are the result of the interference of two vortices with topological charges $\ell$ and $\ell-2$ for $\hat{\mathbf{e}}_+$ and $\ell$ and $\ell+2$ for $\hat{\mathbf{e}}_-$, while the

longitudinal component results from the interference of two vortices with topological charges $\ell+1$ and $\ell-1$. The total energy is proportional to the intensity of these three components, which can be expressed by three terms each:

$$\begin{aligned}
\frac{\mathrm{I}_+}{\mathrm{I}_0} &= \cos^2\beta(|A_\ell|^2p^2+|B_{\ell-2}|^2q^2+2Re\{A_\ell B^*_{\ell-2}pq\mathrm{e}^{2\mathrm{i}\varphi}\}),\\
\frac{\mathrm{I}_-}{\mathrm{I}_0} &= \cos^2\beta(|A_\ell|^2q^2+|B_{\ell+2}|^2p^2+2Re\{A_\ell B^*_{\ell+2}pq\mathrm{e}^{-2\mathrm{i}\varphi}\}),\\
\frac{\mathrm{I}_z}{\mathrm{I}_0} &= 2\cos^2\beta(|C_{\ell+1}|^2p^2+|C_{\ell-1}|^2q^2+2Re\{C_{\ell+1}C^*_{\ell-1}pq\mathrm{e}^{2\mathrm{i}\varphi}\}),
\end{aligned} \tag{14}$$

with

$$\mathrm{I}_0 = |W|^2.$$

Estimating these terms numerically it is easy to see that the leading terms with the weight $\approx 70\%-80\%$ are proportional to $|A_\ell|^2$, interfering third terms are very small in comparison to the others, at least for the experimental conditions we have ($NA = 1.2$). This is very important, since the gradient force, that attracts the particle to the radial stable position, is mainly given by the intensity. We can say that the particle will be affected by the force coming from two main vortices with topological charge $\ell$ and four weaker vortices with topological charges $\ell+1$, $\ell-1$, $\ell+2$, $\ell-2$ owing to the spin-to-orbit conversion.

## Force description - Rayleigh approximation

However such field distribution can be detected only with a local probe either using scattering[11] or velocity of a nanoparticle. Following the approach of a Rayleigh particle, i.e. the particle is much smaller that the wavelength which is treated as the induced dipole. The force components acting upon such nanoparticle in the cylindrical system of coordinates $(r,\varphi,z)$ can be expressed as[14,15]

$$F_r = \frac{1}{2}\Re\left(\alpha_{SI}E_j\,\partial_r E_j^*\right), \tag{15}$$

$$F_\varphi = \frac{1}{2r}\Re\left(\alpha_{SI}E_j\,\partial_\varphi E_j^*\right), \tag{16}$$

$$F_z = \frac{1}{2}\Re\left(\alpha_{SI}E_j\,\partial_z E_j^*\right), \tag{17}$$

where $j=\pm,z$ correspond to the field components in circular basis $\mathbf{E}(r,\varphi,z)=E_+\hat{\mathbf{e}}_++E_-\hat{\mathbf{e}}_-+E_z\hat{\mathbf{e}}_z$, which is related to the field components in cylindrical system of coordinates as:

$$\begin{pmatrix}E_+\\E_-\\E_z\end{pmatrix} = \frac{1}{\sqrt{2}}\begin{pmatrix}\mathrm{e}^{-\mathrm{i}\varphi} & -\mathrm{i}\mathrm{e}^{-\mathrm{i}\varphi} & 0\\ \mathrm{e}^{+\mathrm{i}\varphi} & +\mathrm{i}\mathrm{e}^{+\mathrm{i}\varphi} & 0\\ 0 & 0 & \sqrt{2}\end{pmatrix}\begin{pmatrix}E_r\\E_\varphi\\E_z\end{pmatrix}, \tag{18}$$

and $\alpha_{SI}$ is a polarizability of the particle in SI units, expressed with non-dimensional form $\alpha_0$, and $\alpha_R$ with included radiation reaction term[16], :

$$\alpha = \frac{4\pi\varepsilon_m\varepsilon_0}{k^3}\alpha_R, \qquad \alpha_R = \frac{\alpha_{CM}}{1-\mathrm{i}\frac{2}{3}\alpha_0}, \qquad \alpha_{CM} = (ka)^3\frac{m^2-1}{m^2+2}, \tag{19}$$

where relative refractive index is denoted as $m = n_p/n_m$, $\varepsilon_m = n_m^2$, $\varepsilon_0$ is permittivity of vacuum.

Expressing the azimuthal force using Eq. (13) one would obtain:

$$F_\varphi = \frac{|W|^2}{2r}\Re\left\{\mathrm{i}(\alpha'_{SI}-\mathrm{i}\alpha''_{SI})(p^2A^\varphi_{\ell+}+q^2A^\varphi_{\ell-}+pqA^\varphi_{\ell z})\right\} \tag{20}$$

where the amplitudes were denoted:

$$A^\varphi_{\ell\pm}(r,z) = \ell|A_\ell|^2+2(\ell\pm1)|C_{\ell\pm1}|^2+(\ell\pm2)|B_{\ell\pm2}|^2 \tag{21}$$

$$\begin{aligned}
A^\varphi_{\ell z}(r,\varphi,z) &= \mathrm{e}^{-2\mathrm{i}\varphi}[(\ell-2)A^*_\ell B_{\ell-2}-2(\ell-1)C^*_{\ell+1}C_{\ell-1}+\ell B^*_{\ell+2}A_\ell]\\
&+ \mathrm{e}^{+2\mathrm{i}\varphi}[(\ell+2)A^*_\ell B_{\ell+2}-2(\ell+1)C^*_{\ell-1}C_{\ell+1}+\ell B^*_{\ell-2}A_\ell].
\end{aligned} \tag{22}$$

To express $F_r, F_z$ we use of the fact, that the derivative $\partial_z E$ acts only on the exponential factors in the integrand of $A_m$, $B_m$, $C_m$, which yields extra factor $\mathrm{i}k\cos\theta$. In similar way the derivative $\partial_r E$ affects only the Bessel functions. In analogy, we define other two sets of integrals

$$kX^r_m(r,z) = \frac{\partial X_m(r,z)}{\partial r} \tag{23}$$

$$\mathrm{i}kX^z_m(r,z) = \frac{\partial X_m(r,z)}{\partial z}, \tag{24}$$

where $X_m$ should be substituted with $A_m$, $B_m$, $C_m$:

$$A^r_{\ell\pm}(r,z) = A^*_\ell A^r_\ell + 2C^*_{\ell\pm1}C^r_{\ell\pm1} + B^*_{\ell\pm2}B^r_{\ell\pm2} \tag{25}$$

$$A^r_{\ell z}(r,\varphi,z) = [A^*_\ell B^r_{\ell-2} - 2C^*_{\ell+1}C^r_{\ell-1} + B^*_{\ell+2}A^r_\ell]\mathrm{e}^{-2\mathrm{i}\varphi} + [A^r_\ell B^*_{\ell-2} - 2C^*_{\ell-1}C^r_{\ell+1} + B^r_{\ell+2}A^*_\ell]\mathrm{e}^{+2\mathrm{i}\varphi}. \tag{26}$$

The expressions for $A^z_{\pm0}$ are identical, with the upper index $r$ exchanged for $z$ in each term.

$$F_r = \frac{k|W|^2}{2}\Re\left\{(\alpha'_{SI} - \mathrm{i}\alpha''_{SI})\left(p^2 A^r_{\ell+} + q^2 A^r_{\ell-} + pqA^r_{\ell z}\right)\right\} \tag{27}$$

$$F_z = \frac{k|W|^2}{2}\Re\left\{\mathrm{i}\,(\alpha''_{SI} + \mathrm{i}\alpha'_{SI})\left(p^2 A^z_{\ell+} + q^2 A^z_{\ell-} + pqA^z_{\ell z}\right)\right\} \tag{28}$$

When the paraxial beam incident on the lens is circularly polarized, the expression reduces significantly:

$$F^\pm_\varphi = \frac{|W|^2}{2r}\alpha''_{SI}\left\{\ell|A_\ell|^2 + 2(\ell\pm1)|C_{\ell\pm1}|^2 + (\ell\pm2)|B_{\ell\pm2}|^2\right\} \tag{29}$$

$$F^\pm_r = \frac{k|W|^2}{2}\Re\left\{(\alpha'_{SI} - \mathrm{i}\alpha''_{SI})A^r_{\ell\pm}\right\} \tag{30}$$

$$F^\pm_z = \frac{k|W|^2}{2}\Re\left\{(\alpha''_{SI} + \mathrm{i}\alpha'_{SI})A^z_{\ell\pm}\right\} \tag{31}$$

Analyzing $F_r$ we found that the radial position of the particle depends on the RH or LH circular polarization $\pm\sigma$ of the beam with respect to the topological charge $\ell$. Thus detecting the radial particle position one should detect the spin-orbital coupling of the focused vortex beam, as we show below. Similarly the axial force $F_z$ changes its magnitude with the beam polarization, however, we can not detect these dependence in our experimental arrangement. Looking at $F^\pm_\varphi$ one discovers that this force is fully non-conservative for RH or LH polarization and for paraxial beam it is proportional to $\ell\pm\sigma$[17,18], however, force magnitude increases with stronger non-paraxiality of the beam. Thus detecting the mean time of the particle orbit $T = 2\pi r\gamma/F_\varphi$, where $\gamma$ is the hydrodynamic drag coefficient, one should be also able to detect the spin-orbit interaction, too.

## Estimate of equilibrium position of orbiting particle

Using Eq. (15) and assuming $\alpha''_{SI} \ll \alpha'_{SI}$ it is that seen the radial force is proportional to the radial gradient of the optical intensity $I_+ + I_- + I_z$ expressed in Eq. (14). This can be interpreted that the particle moves in an interference field of five vortex beams with topological charges $\ell$, $\ell+1$, $\ell-1$, $\ell+2$, $\ell-2$ represented by terms $A_\ell$, $C_{\ell\pm1}$, and $B_{\ell\pm2}$, respectively. Let us further assume that in radial direction the particle settles in the equilibrium position $R_{eq}$ and each vortex beam has intensity maximum at radial distance

$$R_\ell \approx m\ell + \delta, \tag{32}$$

assuming for simplicity the same $\delta$ for all $\ell$. This linear behavior can be seen in the data plotted in Fig. 3 for large values of $\ell$. We can write for the radial force, assuming optical trap of each vortex as the Hookian spring with stiffness $\kappa_\ell$

$$F_r(R_{eq}) \approx -2\kappa_\ell(R_{eq} - R_\ell) - \kappa_{\ell+1}(R_{eq} - R_{\ell+1}) - \kappa_{\ell-1}(R_{eq} - R_{\ell-1}) - \kappa_{\ell+2}(R_{eq} - R_{\ell+2}) - \kappa_{\ell-2}(R_{eq} - R_{\ell-2}) \equiv 0. \tag{33}$$

Solving for $R_{eq}$, utilizing Eq. (32) and direct proportionality between trap stiffness and $\kappa_\ell$, $\kappa_{\ell\pm2}$, $\kappa_{\ell\pm1}$ and corresponding terms $|A_\ell|^2$, $|B_{\ell\pm2}|^2$ and $|C_{\ell\pm1}|^2$ in Eq. (14) one obtains

$$R_{eq} \approx m\ell + \frac{\kappa_{\ell+1} - \kappa_{\ell-1} + 2\kappa_{\ell+2} - 2\kappa_{\ell-2}}{2\kappa_\ell + \kappa_{\ell-1} + \kappa_{\ell+1} + \kappa_{\ell-2} + \kappa_{\ell+2}} + \delta. \tag{34}$$

The second term represents a sum of four terms and each of them is proportional to the ratio of the optical intensity in the corresponding vortex to the total intensity $\mathrm{I}_0$ in the beam. Utilizing significant terms in Eq. (14) one ends with

$$R_{eq} \approx m\ell + 2\cos^2\beta\left(|C_{\ell+1}|^2 p^2 - |C_{\ell-1}|^2 q^2 + |B_{\ell+2}|^2 p^2 - |B_{\ell-2}|^2 q^2\right) + \delta. \tag{35}$$

leading directly to Eq. (1) assuming in the first approximation $|C_{\ell+1}|^2 + |B_{\ell+2}|^2 \approx |C_{\ell-1}|^2| + |B_{\ell-2}|^2$. All the applied assumptions are better satisfied for larger topological charges $\ell$, which is also demonstrated in Fig. 5.

### Force description - Generalized Mie approach

However the above mentioned analytical equations are not fully valid for larger particles used in the experiment. Therefore our calculations of the optical force field are based on the exact wave solution in spherical coordinates without applying any small/large particle approximations. Entering the force formula given in Ref.[19] multipole expansion coefficients for the incident field and for the scattered one, (referred to as the 'beam-shape' coefficients[20]) are determined as projections of the (spherical) radial components of the beam field on a spherical harmonic function $Y_n^m(\theta,\varphi)$

$$N_{p,nm}^{(1)} = \frac{1}{\sqrt{n(n+1)}}\left(k^{-1}\nabla\times\right)^p\left(\mathbf{r}\; j_n^{(1)}(kr)Y_n^m(\mathbf{r})\right), \tag{36}$$

where $j_n^{(1)}$ being the spherical Bessel function and the first kind, index $p$ stands for multipole TE ($p=1$) and TM ($p=2$) modes. Extending the procedure to a vortex beam[12] described by Eq. (13) and following[21] we obtained the following expressions

$$a_{p,nm}^{\text{inc}} = \mathrm{e}^{\mathrm{i}(\ell-m)(\varphi+\frac{\pi}{2})}\mathrm{i}^n\, 4\pi k f \mathrm{e}^{-\mathrm{i}kf}\sqrt{\frac{2\pi}{n(n+1)}}\left\{\left[v_2,-\mathrm{i}v_1\right]\cdot(\sigma_2)^p\cdot R_z(-\varphi_0)\cdot\begin{bmatrix}E_0^x\\E_0^y\end{bmatrix}\right\}, \tag{37}$$

where other functions $v_1, v_2$ are given also as integrals over aperture angle $\alpha$:

$$\begin{Bmatrix}v_1\\v_2\end{Bmatrix}_{nm} = \int_0^{\alpha_{max}}(\mathrm{d}\alpha\sin\alpha)(\cos\alpha)^{\frac{1}{2}}A(\alpha)\mathrm{e}^{-\mathrm{i}kz_0\cos\alpha}\begin{Bmatrix}J_{m-\ell}^{+}(kr_0\sin\alpha)\pi_n^m(\alpha)+J_{m-\ell}^{-}(kr_0\sin\alpha)\tau_n^m(\alpha)\\J_{m-\ell}^{-}(kr_0\sin\alpha)\pi_n^m(\alpha)+J_{m-\ell}^{+}(kr_0\sin\alpha)\tau_n^m(\alpha)\end{Bmatrix}. \tag{38}$$

The shortcuts $J_m^{\pm}$ denote

$$J_m^{+}(\xi) \equiv \frac{1}{2}[J_{m-1}+J_{m+1}(\xi)] = \frac{m}{\xi}J_m(\xi) \tag{39}$$

$$J_m^{-}(\xi) \equiv \frac{1}{2}[J_{m-1}-J_{m+1}(\xi)] = \partial_\xi J_m(\xi) \tag{40}$$

Pauli matrix $\sigma_2 = \begin{pmatrix}0 & -\mathrm{i}\\ \mathrm{i} & 0\end{pmatrix}$, $(E_0^x, E_0^y)$ is the Jones vector of the incident field (i.e. $1/\sqrt{2}(1,\pm\mathrm{i})$ for RH/LH polarization) , $\pi(\theta) = mY_n^m(\theta)/\sin\theta$, $\tau(\theta)=\partial_\theta Y_n^m(\theta)$, $R(\varphi)$ is the standard 2D rotation matrix.

Employing the equations presented above with the parameters used in the experiment (see Table **A1**) we calculated the forces acting upon the particle placed at plane C, (i.e. at the plane of the beam focus) and determined the radial equilibrium position $R_{eq}$, radius of the maximal field energy density $R_{ed}$, and orbiting frequency $f_0$ for different circular polarizations and topological charges $\ell$ of the beam presented above.

## References


1. Čižmár, T., Mazilu, M. & Dholakia, K. In situ wavefront correction and its application to micromanipulation. *Nat. Photon.* **4**, 388–394 (2010).
2. Wiersma, H. & TD, V. Defocusing of a converging electromagnetic wave by a plane dielectric interface. *J. Opt. Soc. Am. A* **13**, 320–325 (1996).
3. Jákl, P. *et al.* Behaviour of an optically trapped probe approaching a dielectric interface. *J Mod. Opt.* **50**, 1615–1625 (2003).
4. Kasa, I. A cicrcle fitting procedure and its error analysis. *IEEE Transactions on Intrumentation Meas.* 8 – 14 (1976).
5. Tolić-Nørrelykke, I. M., Berg-Sørensen, K. & Flyvbjerg, H. MatLab program for precision calibration of optical tweezers. *Comp. Phys. Commun.* **159**, 225–240 (2004).
6. Berg-Sørensen, K. & Flyvbjerg, H. Power spectrum analysis for optical tweezers. *Rev. Sci. Instrum.* **75**, 594–612 (2004).
7. Happel, J. & Brenner, H. *Low Reynolds number hydrodynamics* (Prentice–Hall, Englewood Cliffs, 1965).
8. Leach, J. *et al.* Comparison of Faxén's correction for a microsphere translating or rotating near a surface. *Phys. Rev. E* **79**, 026301 (2009).
9. Schaffär, E., Nørrelyke, S. F. & Howard, J. Surface Forces and Drag Coefficients of Microspheres near a Plane Surface Measured with Optical Tweezers. *Phys. Rev. E* **23**, 3654–3665 (2007).

**10.** Richards, B. & Wolf, E. Electromagnetic diffraction in optical systems. 2. Structure of the image field in an aplanatic system. *Proc. Royal Soc. Lond. A* **253**, 358–379 (1959).

**11.** Bliokh, K. Y. *et al.* Spin-to-orbital angular momentum conversion in focusing, scattering, and imaging systems. *Opt. Express* **19**, 26132–26149 (2011).

**12.** Török, P. & Munro, P. R. T. The use of Gauss-Laguerre vector beams in STED microscopy. *Opt. Express* **12**, 3605 (2004).

**13.** Novotny, L. & Hecht, B. *Principles of nano-Optics* (Cambridge University Press, Cambridge, 2006).

**14.** Chaumet, P. & Nieto-Vesperinas, M. Time-averaged total force on a dipolar sphere in an electromagnetic field. *Opt. Lett.* **25**, 1065–1067 (2000).

**15.** Čižmár, T., Šiler, M. & Zemánek, P. An optical nanotrap array movable over a milimetre range. *Appl. Phys. B* **84**, 197–203 (2006).

**16.** Draine, B. The discrete-dipole approximation and its application to interstellar graphite grains. *Astrophys. J.* **333**, 848–872 (1988).

**17.** Allen, L., Beijersbergen, M., Spreeuw, R. & Woerdman, J. Orbital angular-momentum of light and the transformation of Laguerre-Gaussian laser modes. *Phys. Rev. A* **45**, 8185–8189 (1992).

**18.** Simpson, N. B., Dholakia, K., Allen, L. & Padgett, M. J. Mechanical equivalence of spin and orbital angular momentum of light: an optical spanner. *Opt. Lett.* **22**, 52–54 (1997).

**19.** Barton, J. P., Alexander, D. R. & Schaub, S. A. Theoretical determination of net radiation force and torque for a spherical particle illuminated by a focused laser beam. *J. Appl. Phys.* **66**, 4594–4602 (1989).

**20.** Gouesbet, G. & Gréhan, G. *Generalized Lorenz-Mie Theories* (Springer, 2011).

**21.** Alvaro Ranha Neves, A. *et al.* Exact partial wave expansion of optical beams with respect to an arbitrary origin. *Opt. Lett.* **31**, 2477 (2006).
